# ASFV pE146L-induced ER remodeling is essential for viral replication

**DOI:** 10.1128/jvi.00834-25

**Published:** 2025-08-06

**Authors:** Yilin Guo, Sai Niu, Xueying Wang, Zixuan Wang, Rui Liang, Yubei Tan, Zhen Fu, Zhelin Su, Juan Xu, Hongjun Chen, Yuejun Shi, Limeng Sun, Guiqing Peng

**Affiliations:** 1 State Key Laboratory of Agricultural Microbiology, College of Veterinary Medicine, Huazhong Agricultural University 627716, Wuhan, China; 2 Key Laboratory of Preventive Veterinary Medicine in Hubei Province, The Cooperative Innovation Center for Sustainable Pig Production 47895, Wuhan, China; 3 Key Laboratory of Animal Biosafety Risk Prevention and Control (North), Ministry of Agriculture and Rural Affairs, P.R. China, Shanghai Veterinary Research Institute, Biosafety Research Center, Chinese Academy of Agricultural Sciences 12661, Shanghai, China; 4 Key Laboratory of Prevention & Control for African Swine Fever and Other Major Pig Diseases, Ministry of Agriculture and Rural Affairs, Wuhan, China; Northwestern University Feinberg School of Medicine, Chicago, Illinois, USA

**Keywords:** African swine fever virus, E146L protein, ER remodeling, virus assembly

## Abstract

African swine fever virus (ASFV) causes a severe hemorrhagic disease, posing a significant threat to the global pig industry. Although the ASFV encodes nearly 200 proteins, the functions of many remain unknown. Here, we identify the inner envelope protein pE146L as essential for ASFV replication. pE146L, localized in the endoplasmic reticulum lumen, induces endoplasmic reticulum (ER) perinuclear aggregation, and its absence disrupts viral factory formation. Mechanistic studies revealed the first high-resolution crystal structure of pE146L-ΔTM. Structural and biochemical analyses revealed that disrupting the intermolecular disulfide bonds of the dimers (C103A) abrogates ER aggregation and impedes viral replication. Furthermore, pE146L binds lipids via a positively charged surface patch, a function critical for replication, suggesting a role in lipid regulation. These findings highlight the multifunctionality of pE146L in ASFV morphogenesis and provide a promising target for anti-ASFV drug development.

**IMPORTANCE:**

African swine fever virus (ASFV) causes a highly lethal infectious disease in swine; however, our understanding of its replication and assembly mechanisms remains limited, which hinders the development of vaccines and drugs. In this study, we identified the uncharacterized pE146L, a protein of the inner envelope that is required for the viral life cycle. Notably, we found that pE146L showed distinct colocalization and the ability to induce noticeable ER aggregation. Moreover, we solved the first high-resolution crystal structure of the extracellular soluble region of pE146L and discovered that it is a lipid-binding protein. Interestingly, structural and biochemical analyses suggest the potentially significant impact of intermolecular disulfide bonds on ER aggregation and viral replication. These results highlight the multifunctionality of ASFV pE146L, providing new insights for the development of specific antiviral drugs.

## INTRODUCTION

African swine fever (ASF) is a highly contagious and lethal disease in domestic and wild pigs, with nearly 100% mortality (1, 2). First reported in Kenya in 1921, ASF was introduced to the Iberian Peninsula in 1960 and remained endemic there until the mid-1990s. Subsequently, the disease expanded its geographic range across Africa, spreading to previously unaffected regions. ASF was introduced to Georgia in the Caucasus in 2007 and subsequently spread to other European countries. In recent years, it emerged in China and Southeast Asia (3–6). African swine fever has a sustained and serious impact on global food security and economies (7–9). Currently, Vietnam has approved two commercialized gene-deleted attenuated live vaccines, ASFV-G-ΔI177L and ASFV-G-ΔMGF, derived from the ASFV Georgia strain (ASFV-G), for the prevention and control of ASF. Although these vaccines effectively protect against the virulent parental ASFV-G strain, their general applicability in ASF management remains to be proven (10–12).

ASF virus (ASFV), the sole member of Asfarviridae, is a large double-stranded DNA virus (2, 13, 14), with a genome spanning 170–193 kbp (15, 16), encoding approximately 200 proteins (17), half of which lack known function (18). The ASFV virion comprises five structural layers, with mass spectrometry identifying 68 viral proteins contributing to its formation (18). The innermost nucleoid houses the viral genome and nucleoproteins, encased by a core shell of proteolytic products from viral polyproteins pp220 and pp62 (19). Surrounding this is the inner lipid envelope, which is derived from the host ER and becomes an icosahedral structure by the progressive assembly of the capsid layer. The outermost envelope is acquired during viral budding from the cytoplasmic membrane (20, 21). Despite these insights, the incomplete understanding of ASFV protein functions hampers vaccine and drug development.

ASFV morphogenesis occurs in a perinuclear region called the viral factory (VF) (21, 22). Early morphogenesis involves small, curled membranes within the VF (23), derived from the ER, that are transformed into precursor membranes to form the viral inner envelope. The ER during viral infection is largely unknown (24–27).

Here, we identify the uncharacterized protein pE146L as a transmembrane protein targeting the ER and demonstrate its critical role in ASFV replication. We show that pE146L mediates ER remodeling to facilitate viral morphogenesis. Moreover, we present the first high-resolution crystal structure of the extracellular soluble region of pE146L, revealing its lipid-binding protein capacity and key residue interactions. Structural analyses also reveal that Cys103 of pE146L mediates intermolecular disulfide bonds, which are essential for ER aggregation and ASFV replication. These findings advance our understanding of ASFV assembly mechanisms and identify pE146L as a promising target for ASFV control.

## RESULTS

### ASFV pE146L induced ER aggregation

**Fig 1:**
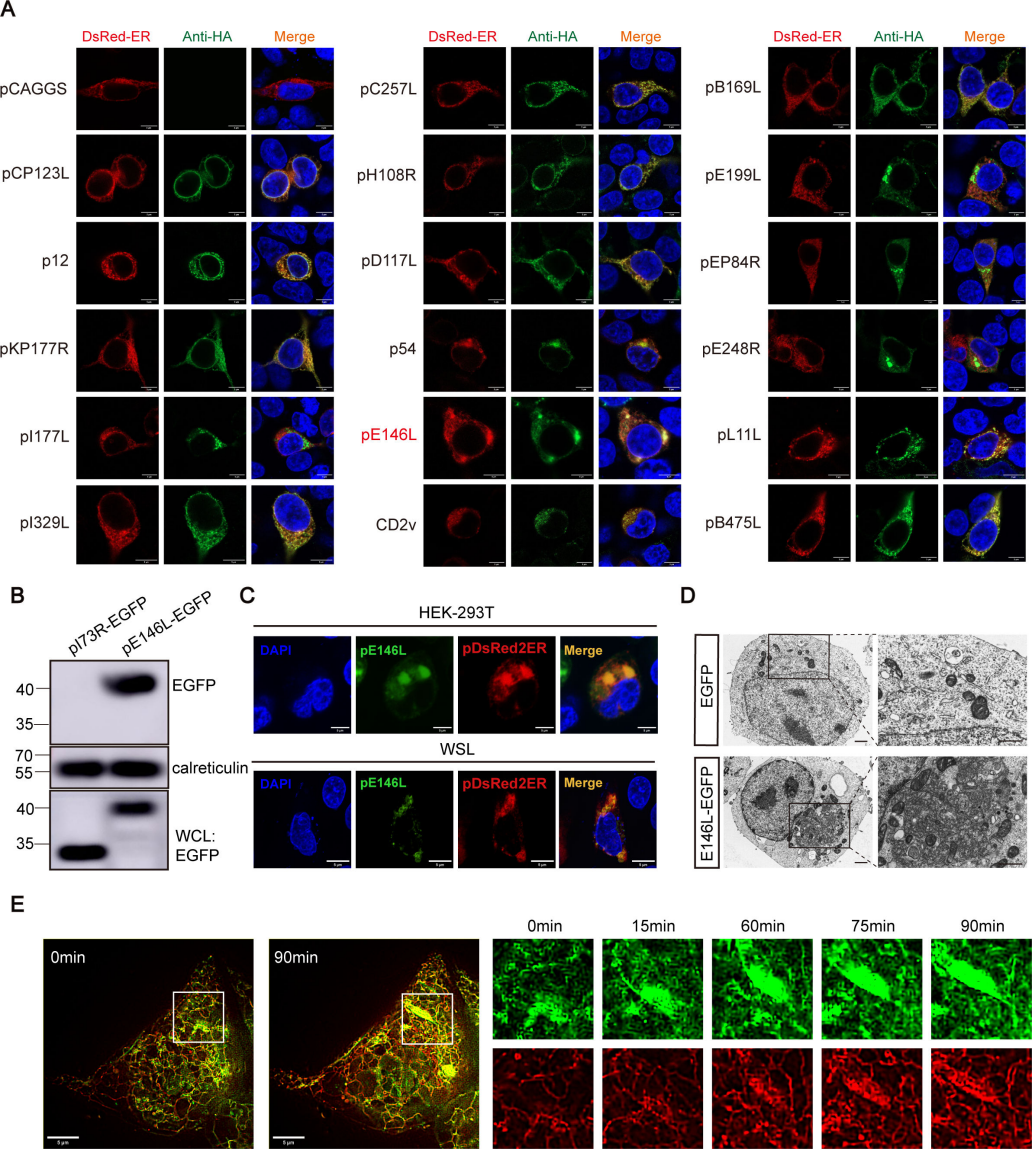
ASFV pE146L Induced ER aggregation. (**A**) 293T cells co-expressing pDsRed2ER (red) and various viral proteins(green) were fixed and stained with HA antibodies 24 hours post-transfection. Scale bar, 5 µm. (**B**) 293T cells transfected with pE146L-EGFP and pI73R-EGFP plasmids were analyzed for ER isolation and Western blotting at 24 hours post-transfection. (**C**) Confocal microscopy showed co-localization of pE146L with the ER in transfected cells. Scale bar, 5 µm. (**D**) Transmission electronmicroscopy (TEM) revealed ER morphology in pE146L-transfected 293T cells. Scalebars, 1 µm as indicated. (**E**) SIM imaging tracked the dynamics of pE146L-EGFP(green) and pDsRed2ER (red) in 293T cells over time. Scale bar, 5 µm.

To identify ASFV proteins capable of inducing ER morphological changes, we co-transfected 293T cells with pDsRed2ER (a marker for ER) and plasmids encoding 17 ASFV proteins with predicted transmembrane domains. Fluorescence imaging revealed that several viral structural proteins exhibited varying degrees of colocalization with the ER. Among them, pE146L and p54 showed similar colocalization and induced noticeable ER aggregation (Fig. 1A).

To confirm this phenomenon of induced ER aggregation, we isolated ER fractions from cells overexpressing pE146L and pI73R using an ER isolation kit. I73R exhibits predominant nuclear localization as established in a prior study (28). Western blot analysis demonstrated significant enrichment of pE146L in the isolated ER fractions (Fig. 1B). To assess the universality of this remodeling effect, we expressed pE146L in both human HEK-293T and porcine WSL cells and observed similar ER aggregation patterns using spatial localization studies (Fig. 1C; Movies S1 and S2). Transmission electron microscopy further validated that pE146L expression in 293T cells caused the ER to form dense perinuclear aggregates, resembling a coiled network encased by mitochondria (Fig. 1D). Time-lapse imaging with structured illumination microscopy (SIM) captured the dynamic nature of the pE146L-induced ER aggregation 16 h post-transfection, pE146L-EGFP expression led to the gradual transition of the ER from elongated strips to clustered aggregates over time. This transformation coincided with the accumulation of pE146L-EGFP (Fig. 1E). These observations suggest that pE146L targets the ER, driving its perinuclear aggregation and remodeling into a compact structure. This dynamic reorganization appears to be consistent with the localization of viral factories during ASFV infection and their characteristic feature of being surrounded by mitochondria (29, 30).

### ASFV pE146L is a late protein of the inner viral envelope

**Fig 2:**
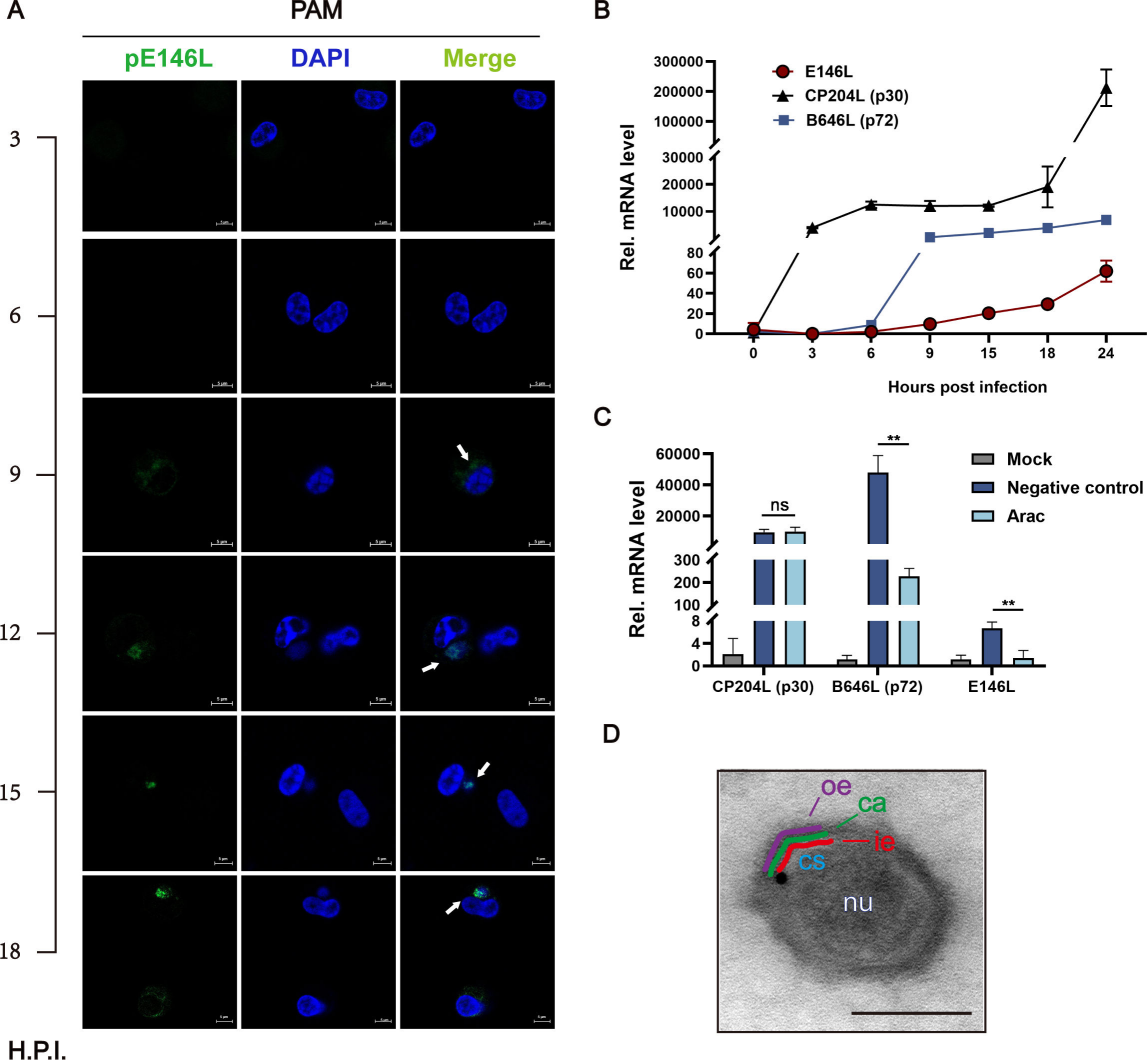
ASFV pE146L is a late protein of the inner viral envelope. (**A**) Primary alveolar macrophages (PAMs) infected with ASFV (MOI = 1) were stained for pE146L (green) and nuclei (blue) at different time points (63×). Scale bar, 5 µm. (**B**) RT-qPCR analysis of E146L gene expression in ASFV-infected PAM cells at various time points. Expression of *CP204L* (p30) and *B646L* (p72) was also measured as controls. (**C**) PAM cells infected with ASFV were treated with AraC (50 µg/mL) at 2 h post-infection and analyzed by RT-qPCR. The means and SDs of the results from three independent experiments are shown. *P < 0.05, **P < 0.01, ***P < 0.001, ns, not significant. *P*-values were determined by two-tailed unpaired Student’s *t*-tests. (**D**) IEM analysis revealed subviral localization of pE146L. The figure highlights the mature extracellular virion with gold particles (triangle) marking the nucleoid (nu, white), core shell (cs, blue), inner envelope (ie, red), capsid (ca, green), and outer envelope (oe, purple). Scale bar, 100 nm.

To characterize the temporal expression profile of pE146L, immunofluorescence staining was performed at different time points post-infection. pE146L became detectable in primary PAM cells approximately 9 h post-infection (hpi) and accumulated steadily throughout the infection cycle (Fig. 2A). To investigate the transcription kinetics of *E146L,* PAM cells infected with ASFV at a multiplicity of infection (MOI) of 1 were harvested at 3, 6, 9, 15, 18, and 24 hpi. Quantitative PCR was used to compare *E146L* mRNA levels, alongside early (CP204L) and late (B646L) ASFV genes. The results showed rapid accumulation of *CP204L* mRNA between 0 and 3 hpi. In comparison, *E146L* transcription began around 6 hpi and increased progressively, coinciding with the transcriptional dynamics of *B646L* (Fig. 2B) (31). After treating infected cells with cytarabine (ara-C), a DNA synthesis inhibitor (32), for 24 h of infection, the mRNA levels of *E146L* and the late gene *B646L* were significantly suppressed. In contrast, the early gene *CP204L* remained unaffected (Fig. 2C), providing further confirmation that pE146L is a late protein.

To determine the subviral localization of pE146L as a structural component of virions, immunoelectron microscopy (IEM) was conducted on ASFV-infected PAM cells at 18 hpi. Gold labeling indicated that pE146L localized primarily to the space between the capsid and the core shell, aligning with the lipid bilayer of the inner viral envelope (Fig. 2D). Altogether, these results indicate that pE146L is an inner envelope protein expressed late in the life cycle of ASFV.

### ASFV pE146L is essential for ASFV replication

To evaluate the role of pE146L in the ASFV life cycle, attempts were made to construct an ASFV mutant lacking *E146L* (28). Despite multiple rounds of purification, only a mixed population of wild-type viruses could be obtained, suggesting that ASFV cannot propagate without *E146L*.

**Fig 3:**
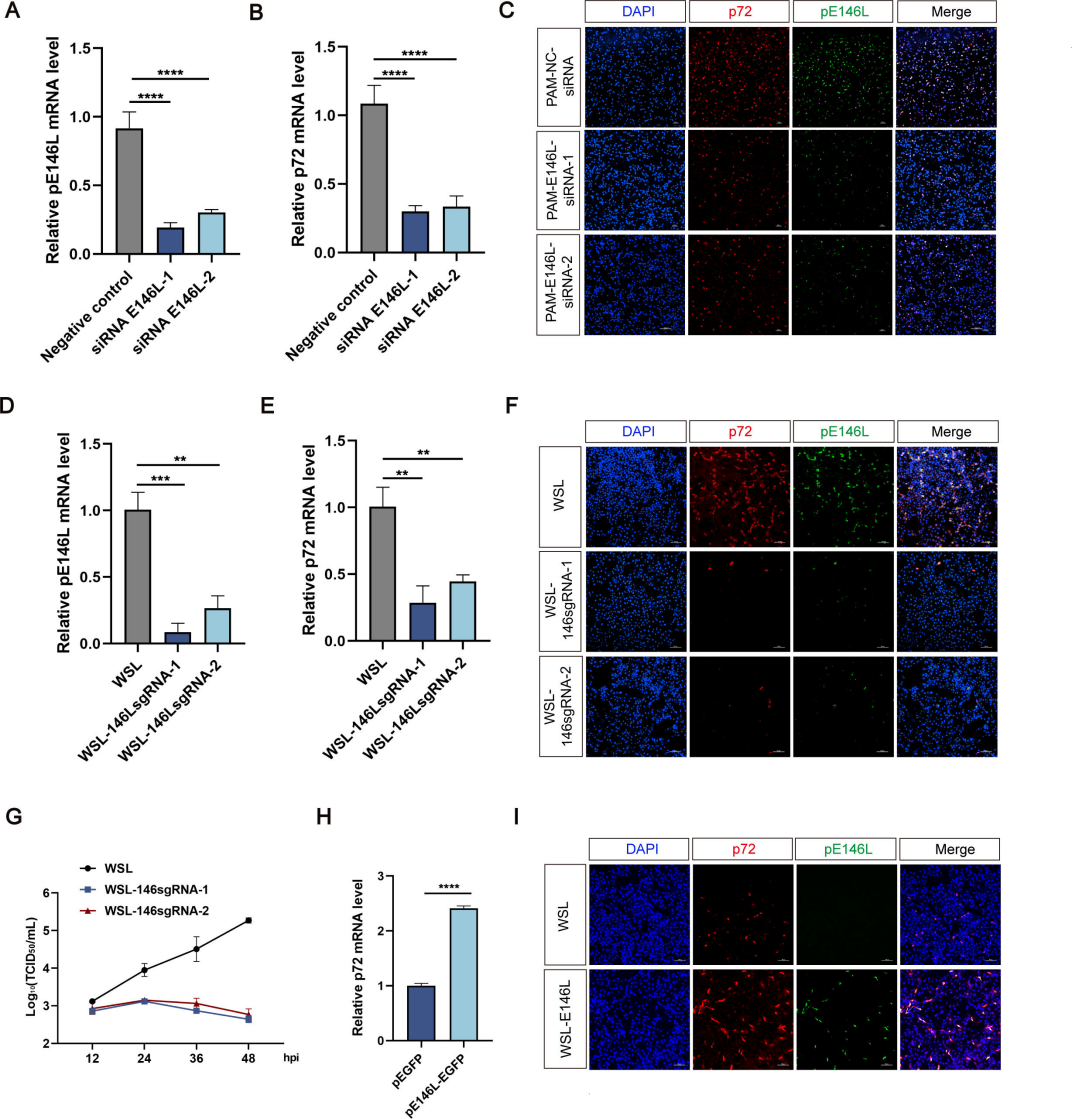
ASFV pE146L is essential for ASFV replication. (**A and B**) PAMs transfected with negative control (NC) siRNA or two E146L-specific siRNAs were infected with ASFV (MOI = 0.1). The mRNA expression of pE146L and p72 was measured by RT-qPCR at 24 hpi. (**C**) Immunofluorescence of pE146L and p72 protein in siRNA-treated PAMs infected with ASFV (MOI = 0.1) at 24 hpi. Scale bar, 100 µm. (**D and E**) Infected wild-type WSL and WSL-E146L-sgRNA cells were analyzed for mRNA expression of pE146L and p72 with ASFV (MOI = 0.1) by RT-qPCR at 24 hpi. (**F**) Immunofluorescence of pE146L and p72 protein in WSL and WSL-E146L-sgRNA cells infected with ASFV (MOI = 1) at 24 hpi. Scale bar, 100 µm. (**G**) The viral titers in WSL and WSL-146sgRNA cells infected with ASFV (MOI = 1) were monitored, with the virus titer of each sample quantified at the indicated hpi. (**H and I**) WSL cells were transfected with an empty vector plasmid and a pE146L plasmid, respectively. At 12 h post-transfection, cells were infected with ASFV (MOI = 1) and collected at 36 hpi for RT-qPCR and immunofluorescence detection. Scale bar, 200 µm. The means and SDs of the results from three independent experiments are shown. *P < 0.05, **P < 0.01, ***P < 0.001, ns, not significant. *P*-values were determined by two-tailed unpaired Student’s *t*-tests.

To further investigate its role, PAM cells were transfected with two distinct small interfering RNAs (siRNAs) targeting *E146L*. Knockdown efficiency was validated by qPCR (Fig. 3A). The mRNA expression of p72 was detected to assess ASFV replication simultaneously (Fig. 3B). This finding coincides with the result of immunofluorescence assays showing that the expression of pE146L accompanied by the concurrent downregulation of p72 in PAM cells transfected with siRNA E146L reduced following ASFV infection (Fig. 3C). To confirm these findings, CRISPR-Cas9 technology was employed to generate WSL cells stably expressing Cas9 and a small guide RNA targeting *E146L* (WSL-146sgRNA). We have conducted comprehensive validation experiments using two independent sgRNAs with unique target sequences to avoid potential off-target effects. Upon ASFV infection, expression levels of pE146L and p72 were significantly decreased in WSL-146sgRNA cells compared with controls (Fig. 3D through F). Moreover, analysis of multiple-step viral growth curves from WSL and WSL-146sgRNA cells culture supernatants revealed a significant attenuation in viral replication capacity, as titers were below those of WSL at all time points and were reduced by more than 2.0 log units at 48 hpi (Fig. 3G). Conversely, overexpression of *E146L* enhanced ASFV replication at 36 hpi compared with cells transfected with an empty vector (Fig. 3H and I). These results indicate that *E146L* is indispensable for ASFV replication, as its knockdown severely impairs viral propagation and its overexpression promotes viral growth.

### Knockdown of pE146L inhibited ASFV viral factory formation

**Fig 4:**
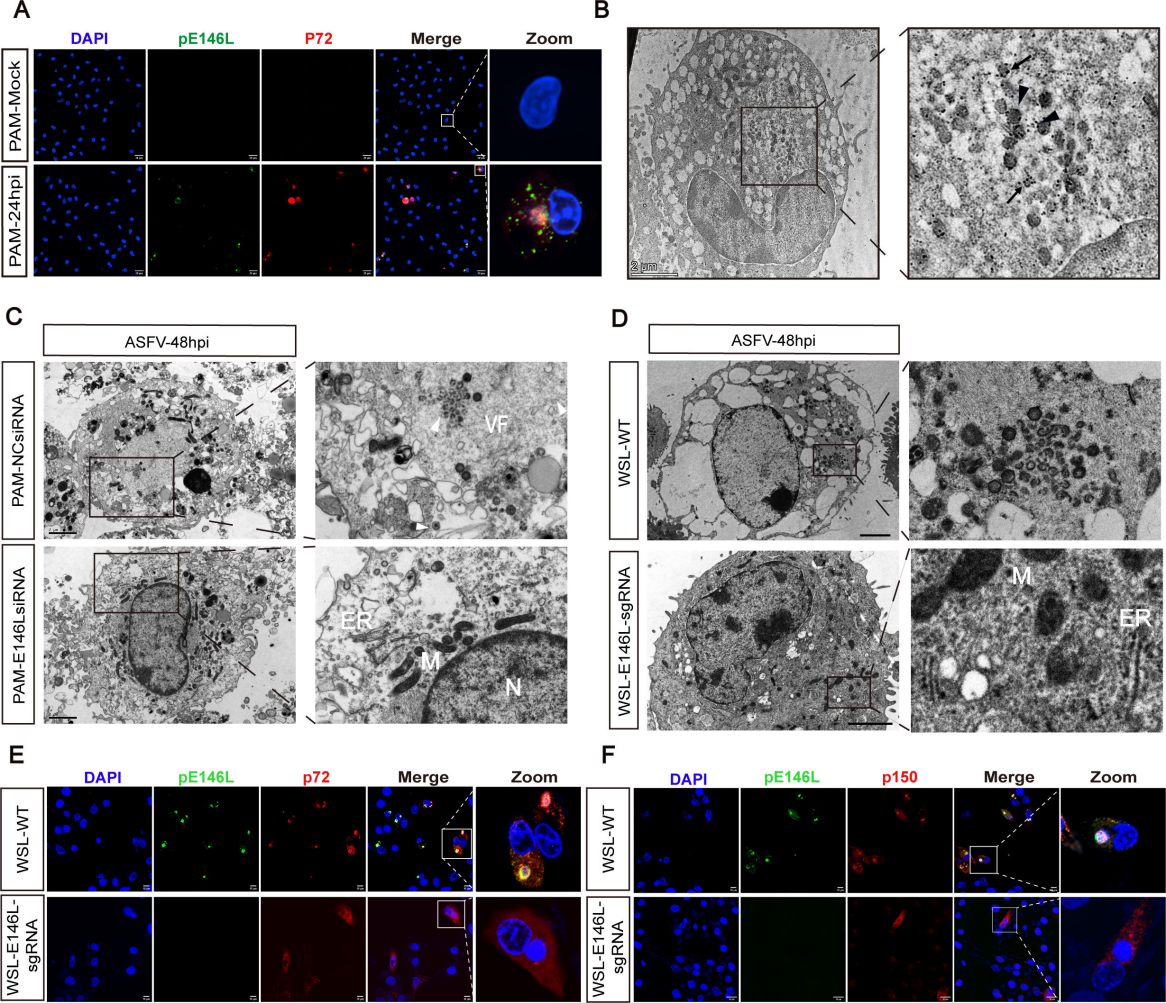
Knockdown of pE146L inhibited ASFV viral factory formation. (**A**) Localization of pE146L in ASFV-infected PAM cells was assessed by immunofluorescence with anti-pE146L (green) and anti-p72 (red) antibodies. Scale bar, 20 µm. (**B**) Immunoelectron microscopy of PAM cells infected with ASFV. Sections of ASFV-infected cells were incubated with anti-pE146L antibodies followed by protein A-gold (10 nm). Triangles indicate the labeling of the icosahedral capsid at the assembly site, and arrows represent the labeling on the curved precursor membrane (Right). Scale bar, 2 µm. (**C and D**) Evaluation of the effects of cells with inhibition of pE146L expression on viral particle assembly by transmission electron microscope. Scale bar, 2 µm. (**E and F**) Subcellular localization of p72 and p150 in WSL cells or WSL-146L-sgRNA cells. Infected cells were fixed at 24 hpi and immunolabeled with rabbit anti-pE146L (green) and mouse anti-p72 (red) or mouse anti-p150 (red). Scale bar, 10 µm.

To explore how pE146L influences viral replication, its subcellular localization was examined in ASFV-infected PAM and WSL cells using immunofluorescence and immunoelectron microscopy. pE146L was predominantly localized to perinuclear viral factories, co-localizing with p72 (Fig. 4A; Fig. S1). Immunoelectron microscopy revealed abundant pE146L labeling within viral factories that were rich in ER-derived precursor membranes and immature virions (Fig. 4B). Given its localization to the viral inner envelope, pE146L was hypothesized to participate in virion assembly. Electron microscopy showed that in cells where pE146L can be normally expressed, viral factories containing precursor membranes and immature virions were observed. However, suppression of *E146L* expression, either by siRNA in PAM cells or CRISPR-Cas9 in the WSL-146sgRNA cells, disrupted virion morphogenesis, with no detectable precursor membranes or immature virions (Fig. 4C and D).

As reported, the core shell protein of p150, hydrolyzed from pp220, is the most abundant virion protein after the main outer capsid p72 (33). Moreover, p72 and p150 are co-localized with viral DNA in the viral factory during infection (18, 19, 34). However, when pE146L expression was suppressed, p72 was dispersed in the cytoplasm (Fig. 4E), whereas p150 showed peripheral localization near perinuclear DNA (Fig. 4F). These findings underscore the essential role of pE146L in viral factory formation and subsequent virion assembly.

### The transmembrane domain of pE146L does not drive ER aggregation

Bioinformatic analysis revealed that pE146L is highly conserved across ASFV genotypes I, II, and recombinants, containing a 21-amino-acid transmembrane (TM) domain at its N-terminus. This domain anchors the protein to the ER, with the C-terminal projecting into the ER lumen (Fig. S2A and B). To evaluate the role of the TM- domain in ER aggregation, 293T cells were co-transfected with either full-length *E146L* or its TM- domain and DsRed2ER. Confocal microscopy revealed that the TM domain alone did not induce ER aggregation, indicating that the C-terminal region is critical for this function (Fig. S2C).

### Structural characterization of ASFV pE146L-ΔTM

**Fig 5:**
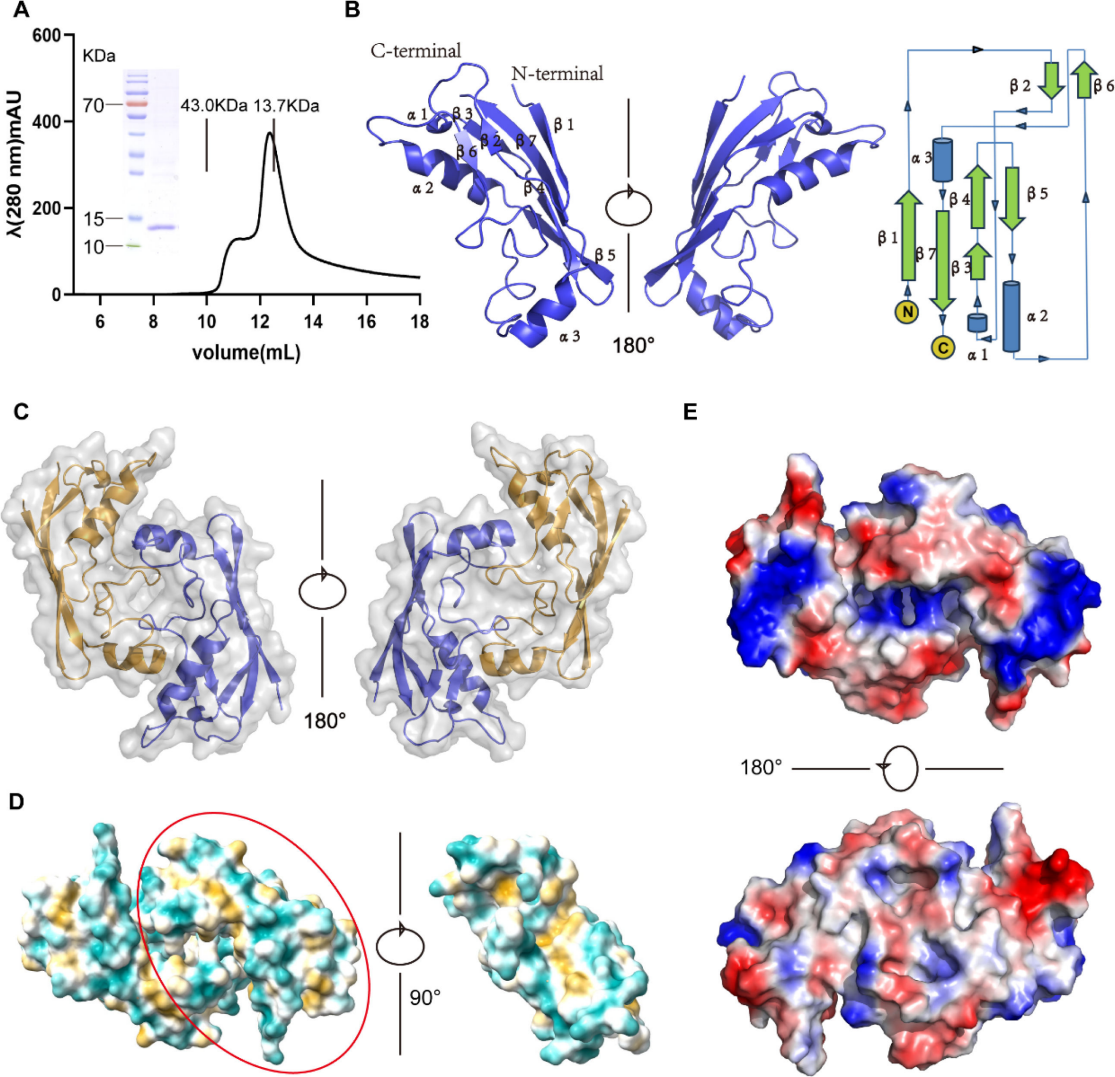
Structural characterization of ASFV pE146L-ΔTM. (**A**) Analytical gel filtration of pE146L-ΔTM. The 280 nm absorbance curve from the Superdex 75 10/300 Gl column and the SDS-PAGE migration profile of the sample is shown. (**B**) Overall structure (PDB ID: 9J46) of pE146L-ΔTM monomer. The monomer structure of pE146L-ΔTM is shown as a cartoon (Left) and a Topology diagram (Right). (**C**) Two subunits of the homodimer are shown (two orientations, rotated by 180°). Different colors indicate different subunits. (**D**) Hydrophobic properties of pE146L-ΔTM. The region of the dimeric interface is shown on the right. Hydrophilic residues are depicted in cyan, and hydrophobic residues are represented in pale yellow, illustrating their spatial distribution within the protein structure. (**E**) Electrostatic properties of ASFV pE146L-ΔTM, Red indicates negatively charged amino acid residues. The positive and negative charges are colored from blue to red with limits ± 58.819 kT/e.

Since pE146L lacks functional motifs or homologs, its potential functions were investigated through structural studies. A truncated version of pE146L lacking the TM domain (pE146L-ΔTM) fused with a C-terminal 6 × His tag was expressed in *Escherichia coli* and purified using Ni-affinity chromatography and gel filtration chromatography. Gel filtration revealed that pE146L-ΔTM predominantly exists as a monomer (~14 kDa), with a minor dimeric fraction (Fig. 5A). Crystals of pE146L-ΔTM grew in the *P*6_5_22 space group and diffracted to a resolution of 2.06 Å. Structural refinement yielded an *R*-_work_ and *R*-_free_ of 21.3% and 25.2%, respectively (Table 1). Except for unobservable residues 65–66, 76–77, and the C-terminal His tag, all residues of the ASFV pE146L-ΔTM were constructed in the final model. The crystal structure of pE146L-ΔTM revealed that the monomer included seven β-strands and three α-helices (Fig. 5B). Based on the subunit interface, pE146L-ΔTM is deposited in the asymmetric unit in antiparallel dimeric form (Fig. 5C). The surface properties of the structure revealed that the surface residues of the protein are mainly hydrophilic. Some hydrophobic residues are distributed in the cavity of the dimer interface (Fig. 5D). In addition, regions with dense distribution of positive charge symmetry were also identified on both sides of the structure (Fig. 5E). A structural similarity search using the DALI server revealed that it had no structural homology with the already identified protein, suggesting that pE146L adopts a novel fold.

**TABLE 1:** X-ray data collection and refinement statistic^*a*^

Parameter	Value for ASFV pE146L-ΔTM
Data collection statistics	
Wavelength (Å)	0.97918
Space group	P6_5_22
Unit cell	
a, b, c	75.9 Å 75.9 Å 570.2
α, β, γ	90.00° 90.00° 90.00°
Resolution (Å)	2.06 Å
Unique reflections	52054 (2765)
Completeness (%)	83.26 (45.79)
Mean I/sigma(I)	29.02 (2.79)
*R*_merge_ (%)^*b*^	7.9 (83.7)
*R*_measure_ (%)^*c*^	8.0 (87.0)
*R*_pim_ (%)^*c*^	1.4 (22.6)
CC_1/2_ (%)	100 (82.9)
Refinement statistics	
Resolution range (Å)	20.8-2.1 (2.13-2.06)
*R*_work_/*R*_free_ (%)^*d*^	21.3/25.2
Protein atoms	5770
Protein residues	708
Ligand atoms	none
Water atoms	6
RMSD	
Bond lengths (Å)	0.009
Bond angles (°)	1.27
Ramachandran plot (%)	
Favored	96.24
Allowed	3.6
Outliers	0.14
PDB code	9J46

^*a*^ Values in parentheses represent the highest resolution shell.

^*b*^ *R*_merge_ = *Σ*_*hkl*_*Σ*_i_|*I(hkl*)_i_-<*I*(*hkl*)>|*/Σ*_*hkl*_Σ_*i*_*I*(*hkl*)i, where *I*(*hkl*) is the intensity of reflection *hkl* and its symmetry equivalents and <*I*(*hkl*)> is the average intensity over all equivalent reflections.

^*c*^ *R*_measure_: multiplicity-weighted *R*_merge_; *R*_pim_: precision-indicating *R*_*merge*_.

^*d*^ *R *= *Σ*||*Fo*| − |*Fc*||/*Σ*|*Fo*|. |*Fo*| and |*Fc*| are amplitudes of the observed and calculated structure factors, respectively. *R*_work_ is the *R* value for reflections used in the refinement, whereas *R*_free_ is the R value for 5% of the reflections, which are selected in thin shells and are not included in the refinement.

### The lipid-binding function of pE146L is essential for ASFV replication

**Fig 6:**
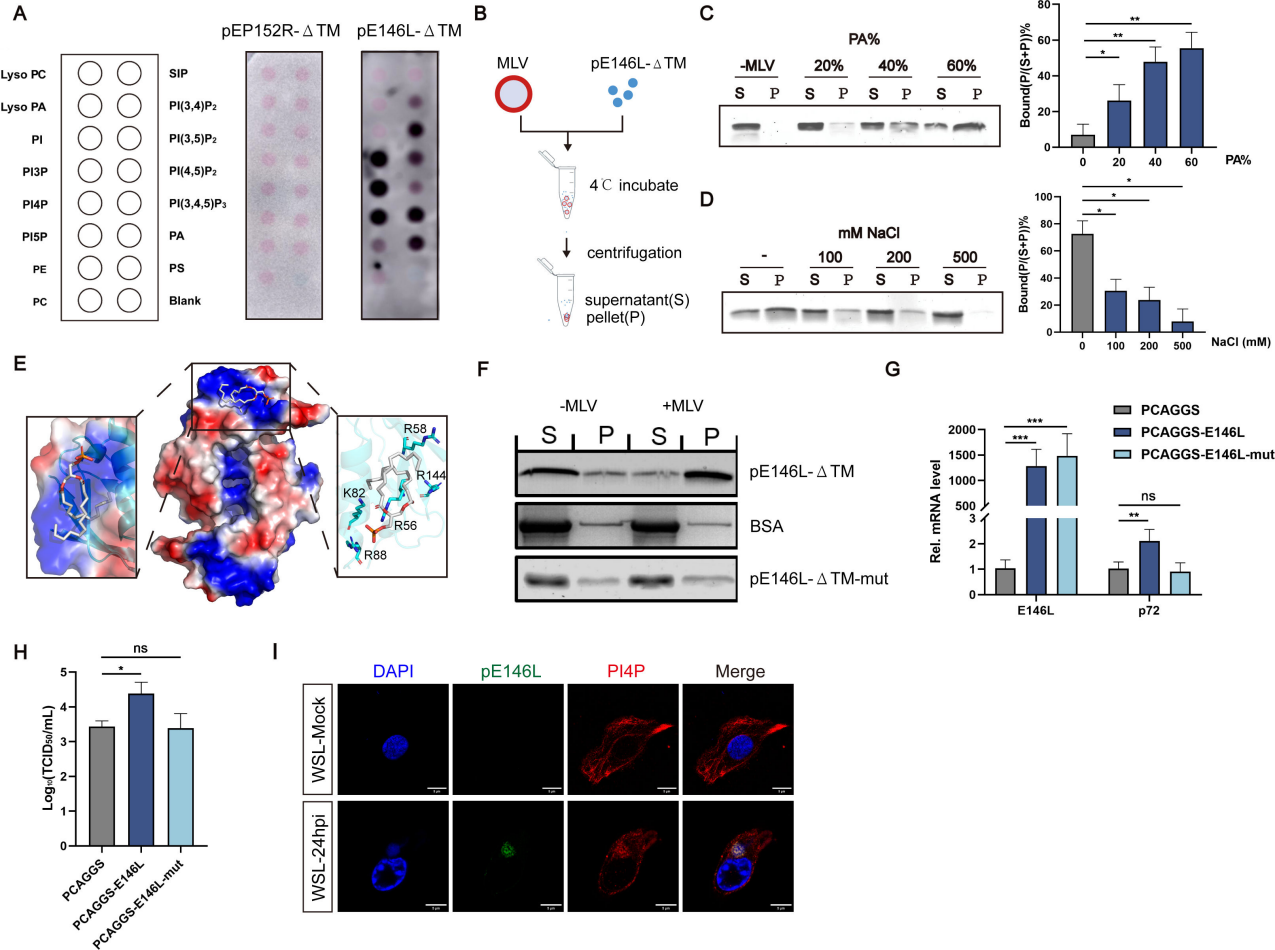
The lipid-binding function of pE146L is essential for ASFV replication. (**A**) Protein-lipid overlay assay. The recombinant pE146L-ΔTM was incubated at 3 mg/mL using commercial lipid arrays. (B) Co-sedimentation assays. Purified pE146L-ΔTM protein was incubated with prepared Multilamellar large vesicles (MLVs) containing different proportions of PA. After high-speed centrifugation, MLVs would be deposited at the bottom of the EP tube. At this time, if there is a binding between protein and lipid vesicles, proteins can be detected in the bottom particle layer (pellet, P) by western blot assay. In contrast, non-interacting proteins are still present in the supernatant of the system (supernatant, S). (**C**) Liposomes are composed of 80% phosphatidylcholine (PC) and 20% phosphatidylic acid (PA) (PA increased accordingly). Supernatant and pellet fractions were separately supplemented with SDS-PAGE loading buffer, subjected to electrophoresis, and visualized by Coomassie Brilliant Blue staining. Densitometric analysis of stained gels was performed to quantify protein distribution (left). The percentage of protein in the supernatant relative to total protein content was quantified and designated as the “bound%” fraction (right). (**D**) Purified pE146L-ΔTM was incubated with MLVs and an elevated concentration of NaCl. SDS-PAGE analysis and the calculation of “bound%” were performed as described in (**C**). (**E**) AutoDock Vina 1.1.2 software was used to perform molecular docking of PA with pE146L-ΔTM. Zoomed-in view of the boxed region showing the amino acid composition of the interaction region. (**F**) The binding ability of the mutant protein to lipid was detected via co-sedimentation assays. (**G and H**) WSL cells were transfected with empty and pE146L-mut plasmids, respectively. At 12 h post-transfection, all cells were infected with ASFV (MOI = 1). RT-qPCR detected the relative mRNA level of p72 in infected cells, and supernatants were harvested at 48 hpi to detect the viral titer. The means and SD of the results from three independent experiments are shown. *P < 0.05, **P < 0.01, ***P < 0.001. ns, not significant. *P*-values were determined by two-tailed unpaired Student’s *t*-tests. (**I**) Confocal images show changes in PI4P localization before and after ASFV infection. pE146L, anti‐pE146L; PI4P, anti‐PI4P. Scale bar, 5 µm.

A distinct surface patch composed of basic residues was identified, leading to the hypothesis that pE146L-ΔTM binds lipids. To confirm this supposition, we employed a protein–lipid overlay assay and purified a recombinant ER-localized protein pE152R (35, 36) lacking its transmembrane region (pE152R-ΔTM) to serve as an irrelevant control. The result showed that compared with pE152R-ΔTM, purified pE146L-ΔTM interacts with acidic lipids to varying degrees, including phosphatidic acid (PA), phosphatidylserine (PS), and phosphoinositides, with stronger binding to highly charged liposomes (Fig. 6A) (37). Liposome co-sedimentation experiments (Fig. 6B) demonstrated that the membrane binding capacity of pE146L-ΔTM is tied to its lipid composition. As the proportion of acidic lipid PA increases within the incubation system (Fig. 6B), the membrane-binding capacity of pE146L-ΔTM increases accordingly (Fig. 6C). Additionally, co-sedimentation of pE146L-ΔTM with liposomes was sensitive to salt, as the binding effect of protein/liposome could be destroyed with increased salt (Fig. 6D), further demonstrated that pE146L-ΔTM mainly binds to the membrane through electrostatic interaction.

To investigate the potential targets of pE146L-ΔTM interacting with lipids, molecular docking analysis was performed using AutoDock Vina software. The final docked 3D models of the crystal structure of pE146L-ΔTM and the ligand PA are shown (Fig. 6E). We found that PA primarily binds to the positively charged patch on the protein surface. Charge distribution analysis identified a positively charged surface region composed of residues R88, K82, R56, R58, and R144. Alanine substitutions of these residues significantly reduced lipid-binding ability, as shown by liposome co-sedimentation experiments (Fig. 6F). Loss of lipid-binding function impaired ASFV replication, as demonstrated by reduced relative transcription level of p72 and viral titers (Fig. 6G and H). Interestingly, phosphoinositol PI4P, one of the lipids that E146L binds, was recruited to viral factories during infection, suggesting that lipid-binding activity may contribute to the formation and function of viral factories (Fig. 6I). To assess whether lipid-binding induces ER remodeling, confocal microscopy and TEM were performed in 293T cells transfected with lipid-binding-deficient mutants (pE146L-mut). Perinuclear ER aggregates were still observed, indicating that lipid binding is not essential for ER remodeling (Fig. S3).

### pE146L induces endoplasmic reticulum aggregation through disulfide bonds to promote viral replication

**Fig 7:**
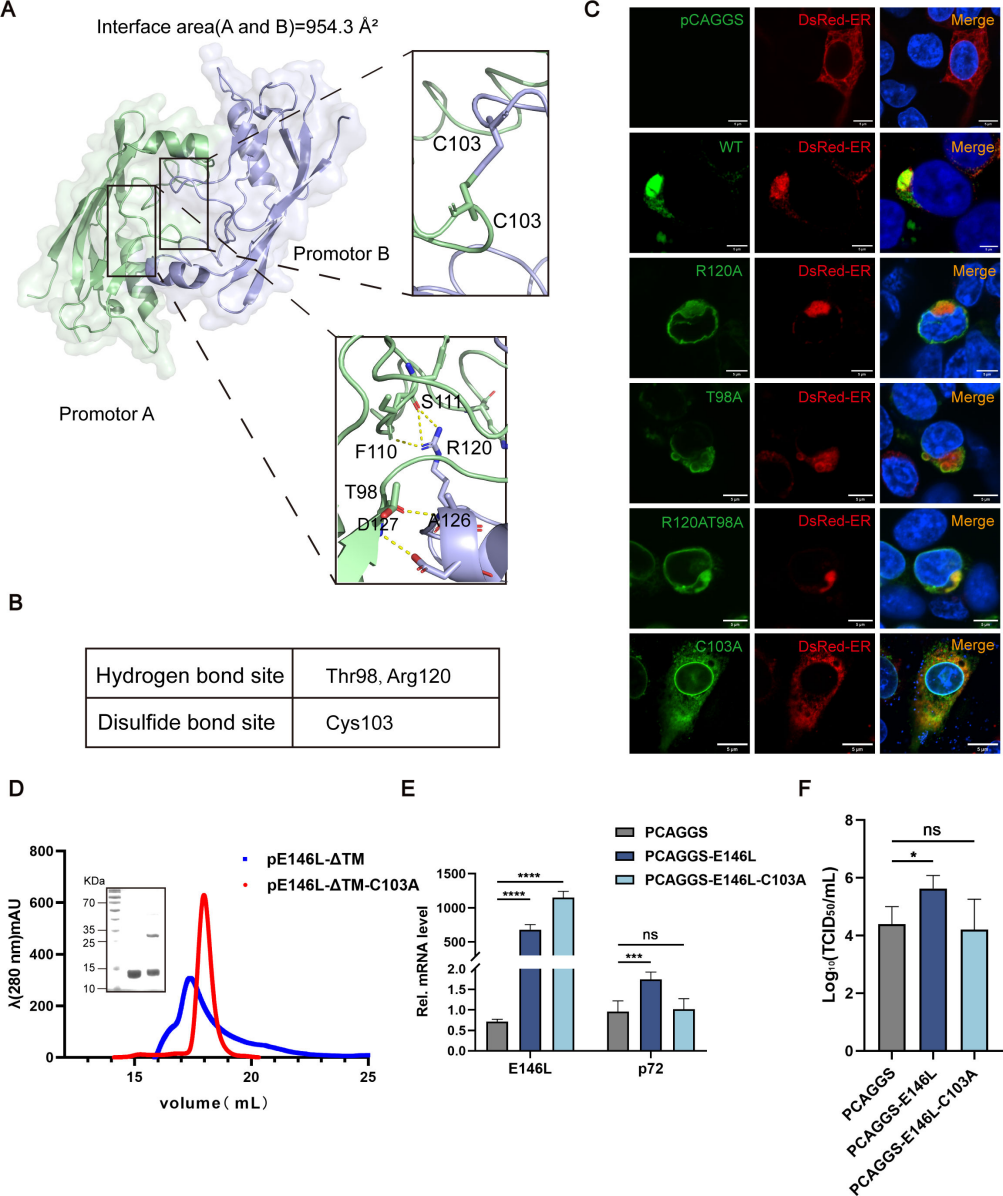
pE146L induces ER aggregation through disulfide bonds to promote viral replication. (**A**) Ribbon diagram of the pE146L-ΔTM dimer. A zoomed-in view of the black-boxed region shows the hydrogen bond and disulfide bond network. (**B**) Residues involved in the dimeric interface. (**C**) Immunofluorescence of intracellular pE146L and indicated mutants. 293T cells co-transfected with plasmids encoding pE146L-WT or mutants and pDsRed2ER. Scale bars, 5 µm. (**D**) Analytical gel filtration of pE146L-ΔTM and pE146L-ΔTM-C103A. The 280 nm absorbance curve from the Superdex 200 Increase 10/300 Gl column and the SDS-PAGE migration profile of the sample are shown. (**E**) RT-qPCR assay of the relative mRNA level of p72 in cells transfected with different plasmids at 48 hpi. (**F**) Quantification of virus infectivity (TCID_50_) in culture supernatant collected 48 hpi from ASFV-infected (MOI = 1) WSL cells transfected with plasmids encoding pE146L-WT, pE146L-C103A, or empty. The means and SD of the results from three independent experiments are shown. *P < 0.05, **P < 0.01, ***P < 0.001, ****P < 0.0001. ns, not significant. *P*-values were determined by two-tailed unpaired Student’s *t*-tests.

Analyzing the structure of pE146L-ΔTM to evaluate the role of specific residues that play an important role in ER aggregation, the results revealed homodimer formation mediated by intermolecular disulfide bonds. The major molecular interface, calculated using the PISA server, was 954.3 Å² in area (Fig. 7A). The key interactions included hydrogen bonds between Thr98 and Arg120 of the dimeric interface and a disulfide bond involving Cys103 (Fig. 7B). Therefore, alanine substitutions were introduced at Cys103, Arg120, and Thr98. Immunofluorescence analysis in 293T cells showed that mutants R120A, T98A, and R120T98A largely maintained ER aggregation, similar to wild-type pE146L. In contrast, the C103A mutation disrupted ER aggregation, resulting in a more diffuse distribution pattern (Fig. 7C). Functional studies demonstrated that the C103A mutation abolished the promotive effects of pE146L on ASFV replication. WSL cells transfected with wild-type or mutant pE146L plasmids showed that the relative transcription level of p72 (RT-qPCR) and viral titers (TCID_50_) was significantly reduced for C103A mutants (Fig. 7D and E). These results indicate that disulfide bonds formed by Cys103 are critical for ER aggregation and ASFV replication.

## DISCUSSION

In this study, we demonstrated that pE146L is indispensable for ASFV replication using siRNA and sgRNA to knock down its expression in infected cells. Previous attempts to delete the *E146L* gene from the ASFV genome failed, resulting only in a hybrid strain rather than a pure recombinant virus lacking pE146L. This suggests that pE146L performs essential, irreplaceable functions, reinforcing the conclusion that the protein is critical for viral growth. Consistent with our findings, pE146L was identified as an internal envelope protein of the virion. Deletion of other inner envelope proteins, such as p54, p17, and pEP84R, has been shown to cause abnormal virion morphology (23, 38, 39). These results underscore the essential roles of inner envelope proteins during ASFV assembly. Additional evidence links ASFV inner envelope proteins, such as pE248R and pE199L, to membrane fusion and core penetration (40–42). Whether pE146L similarly influences viral invasion or budding remains an open question requiring further study.

As shown above, E146L is a late-expressed protein, which is consistent with previously reported transcriptome data (31). Viral late-expressed proteins are generally considered to be associated with virion assembly, release, and virulence (28, 43). Our data suggest that pE146L is integral to the formation of the viral factory at the assembly stage. When pE146L expression was silenced, neither perinuclear virions nor precursor membranes were observed in WSL-sgRNA or PAM cells, implying that pE146L plays a crucial role in the early stages of viral assembly, even before precursor membranes form. Moreover, in the absence of pE146L, structural proteins such as the major capsid protein p72 and the major core shell protein p150 failed to localize correctly, instead dispersing throughout the cytoplasm. These findings highlight the necessity of pE146L in targeting structural proteins in the viral factory. Interestingly, p54 has been implicated in precursor membrane recruitment, as its inhibition prevents the formation of viral factories, resulting in amorphous electron-dense regions devoid of viral structures, as well as the aberrant accumulation of uncleaved core polyproteins pp220 and pp62 forming zipper-like structures (39). These phenomena suggest that these two ER membrane proteins may exhibit a certain degree of overlapping functions. However, considering that the depletion of each protein is sufficient to cause severe phenotypes, we speculate that one possibility is that the two membrane proteins also have a complementary relationship in function; depleting either protein could hinder the normal formation of viral particles. Future research is needed to elucidate the cooperative mechanisms between pE146L, p54, and other ASFV proteins during precursor membrane formation.

A notable property of pE146L is its ability to induce perinuclear ER aggregation when expressed independently. Despite the weak sequence similarity between pE146L and vaccinia virus protein A17, both proteins induce similar ER remodeling, transforming it into a clustered tubular network resembling a “ball of yarn” (44). A17 is a reticulon-like protein in poxviruses that drives membrane bending, a function essential for viral morphogenesis. Inhibiting A17 synthesis halts viral assembly at the crescent membrane stage (45, 46). Since both ASFV and poxvirus infectious particle assembly occurs in cytoplasmic viral factories and involves ER-derived membranes, this suggests an evolutionary and functional relationship between these viral systems.

Structural analysis revealed that there are intermolecular disulfide bonds at the pE146L dimer interface. Disruption of these bonds abolishes ER aggregation and significantly impairs viral replication, indicating that protein oligomerization may play a pivotal role. Protein oligomerization is a well-documented mechanism for inducing membrane deformation across diverse viral systems. For example, the herpesvirus nuclear exit complex (NEC) forms a hexagonal honeycomb structure through oligomerization, driving membrane deformation during virion budding (47, 48). Similarly, the Ebola virus matrix protein VP40 requires dimerization for proper localization to the plasma membrane; disruption of its dimeric state impairs budding and the formation of virus-like particles (49, 50). These highlight the contribution of protein oligomerization in inducing membrane deformation, suggesting that pE146L oligomerization may facilitate ER aggregation and precursor membrane formation, underscoring its critical role in the viral lifecycle.

In this study, we identified pE146L as a lipid-binding protein through structural and biochemical analyses, demonstrating that its positively charged region is critical for lipid-binding activity. This property is essential for ASFV replication. Sequence analysis of pE146L across different ASFV strains revealed that key lipid-binding residues R55, K85, R88, and R144 are highly conserved. Although R58 undergoes a mutation from arginine to lysine in certain strains, its positive charge is retained, suggesting that electrostatic interactions underpin pE146L’s lipid-binding functionality and play a pivotal role in the ASFV lifecycle.

The importance of lipid-binding in viral replication is consistent with findings from other viral systems. For example, one of the key components of poxvirus viral membrane assembly protein (VMAP) is the H7 protein, which binds to phosphoinositol via surface-exposed basic residues. This feature is essential for poxvirus replication (51). Similarly, the electrostatic interaction of flavivirus NS1 with negatively charged lipids is a key mechanism driving the formation of replication organelles (52). These parallels highlight the conserved role of lipid-binding properties in the lifecycle of diverse viruses.

The endocytic pathway serves as a pivotal stage in the infectious cycle of ASFV, wherein the disassembly of viral particles exhibits a spatiotemporal correlation with the biogenesis of endosomal compartments (53, 54). Interestingly, lipid mediators are crucial for early endosome (EE) maturation and multivesicular body (MVB) biogenesis, among which phosphoinositides (PIs) play an indispensable role. Notably, disrupting the balance of phosphatidylinositol 3-phosphate (PtdIns3P) or phosphatidylinositol 3,5-bisphosphate [PtdIns (3, 5)P₂] would have a negative impact on ASFV infection (55). In addition, ASFV is known to redistribute free cholesterol to viral replication sites (56), whereas its utilization of other lipid types remains poorly understood. Our results indicate that ASFV infection requires the recruitment of phosphoinositide PI4P to the viral factory. However, whether PI4P recruitment is mediated directly by pE146L and what specific role PI4P plays in viral factory dynamics remain to be elucidated.

**Fig 8:**
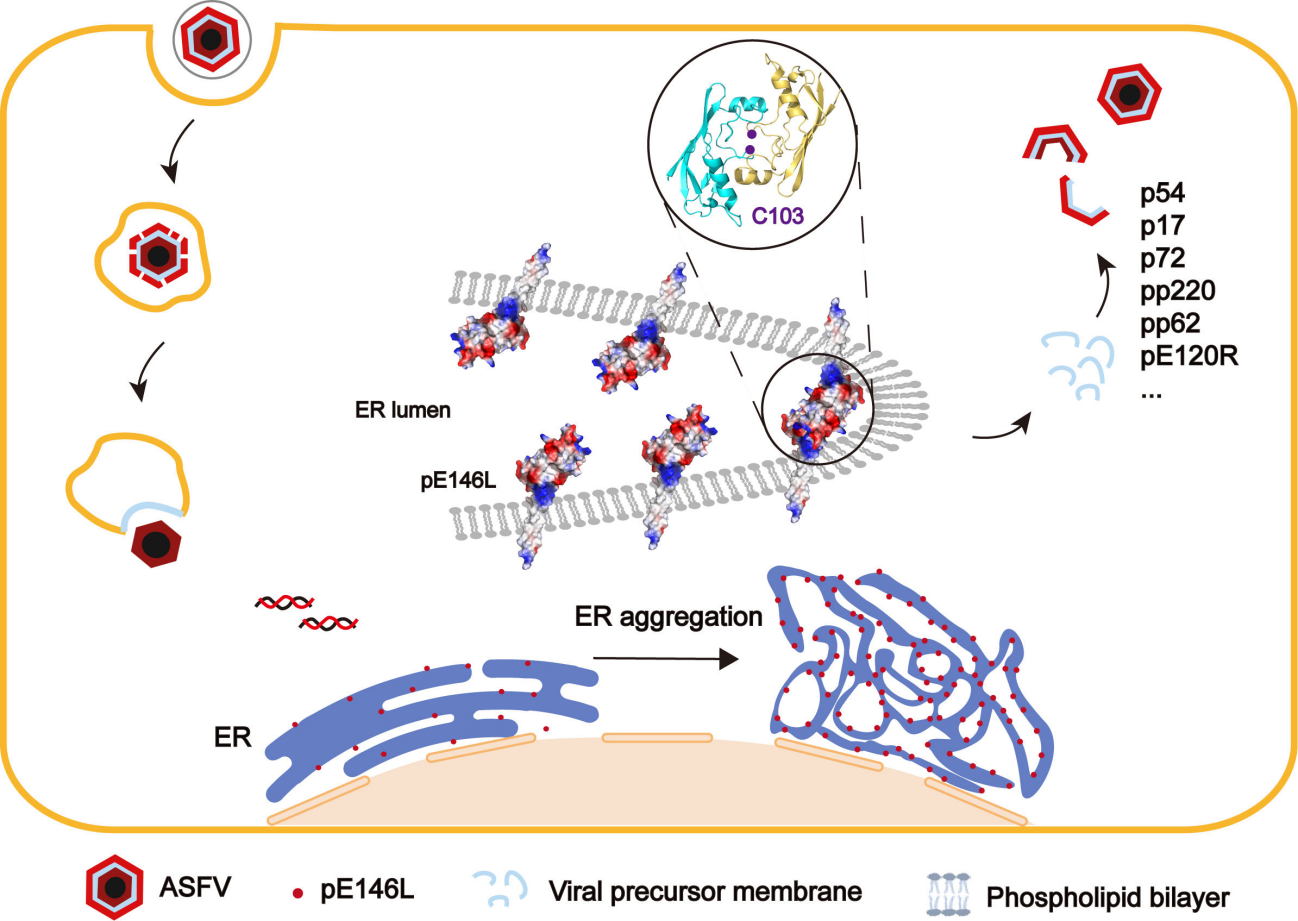
A model illustrating the role of pE146L in the ASFV replication cycle is shown. The transmembrane protein pE146L expressed during the ASFV infection process targets the ER. It triggers the perinuclear aggregation of the ER through intermolecular disulfide bonds, providing membrane materials for the assembly of viral particles.

Based on these findings, we propose a hypothetical model outlining the role of pE146L in the ASFV lifecycle (Fig. 8). pE146L targets the ER through its transmembrane domains and induces perinuclear aggregation of the ER via intermolecular disulfide bonds formed by the C103 residue, thereby supplying membrane materials for virion assembly. Additionally, its lipid-binding properties may facilitate the formation of a viral factory. In summary, our study offers novel insights into ASFV assembly mechanisms, highlighting the crucial role of pE146L in the early stages of morphogenesis. These findings also highlight pE146L as a promising target for developing vaccines or antiviral strategies to disrupt ASFV replication.

## MATERIALS AND METHODS

### Virus and cells

Porcine alveolar macrophages (PAMs) were harvested from the lungs of 4-week-old piglets as described previously. Cells were cultured in RPMI 1640 medium supplemented with 10% fetal bovine serum (FBS), 1% penicillin–streptomycin (Beyotime), and 1% antibiotic-antimycotic (Thermo Fisher Scientific). HEK293T cells (sourced from laboratory stock and stored in liquid nitrogen) were maintained in Dulbecco’s modified Eagle’s medium (DMEM) containing 10%FBS. Wild boar lung (WSL) cells were grown in RPMI 1640 supplemented with 10%FBS. All cell cultures were maintained at 37°C in a humidified incubator with 5%CO_2_. The WSL-146sgRNA-1 and WSL-146sgRNA-2 cell lines we constructed are both monoclonal cell lines obtained by culturing after flow cytometry sorting.

The ASFV strain HB01 has been described before (35). Unless otherwise specified, viral infections were conducted using extracellular particles obtained from infection supernatants, clarified via low-speed centrifugation (1,000 × *g* for 10 min).

### Expression plasmids

The *E146L* gene was amplified by PCR using ASFV genomic DNA as the template. The amplified DNA fragment was cloned into the pET-42b (+) vector treated with restriction enzymes *NdeI* and *XhoI* (NEB), adding a C-terminal hexa-histidine tag. For eukaryotic expression, the *E146L* fragment was cloned into a linearized pCAGGS vector (*EcoRI* and *XhoI* restriction sites; NEB) through homologous recombination. Truncated and mutated E146L constructs were similarly cloned into either pET42b (+) or pCAGGS vectors. To create a lentiviral sgRNA expression vector, paired sgRNA oligonucleotides (sgRNA-1: 5′-GTTGTCTATAACTATTGTGT-3′, sgRNA-2: 5′-CCCCTTGCTGTACTTAAAGG-3′) were annealed and inserted into a lentiCRISPR-v2 vector linearized with *BbsI*. For generating stable pE146L-EGFP overexpression cell lines, *E146L-EGFP* coding sequences were cloned into the pLVX-IRES-Puro vector (*XbaI* and *NotI* sites; NEB). All plasmids were confirmed by sequencing to ensure the absence of undesired mutations. All primer sequences are listed in Table S1.

### Viral titers

PAMs or WSL cells were inoculated with ASFV in 24-well plates in triplicate. The infected cells were harvested, along with their culture supernatants, at the indicated times post-infection, and viral titers were determined based on TCID_50_. Specifically, the collected supernatants were subjected to 10-fold serial dilutions and subsequently added to 96-well plates pre-seeded with PAMs. Following 3–5 days of incubation, the cell plates were processed according to the immunofluorescence assay protocol, in which the p30-specific antibody was used to label infected cells. Viral titers were subsequently observed by fluorescent microscopy and calculated.

### Protein expression and purification

Expression plasmids were transformed into *E. coli* BL21(DE3) cells. Cultures were grown in LB medium containing 50 μg/mL kanamycin at 37 °C to an OD_600_ of 0.6–0.8. Protein expression was induced by adding 0.8 mM IPTG, followed by incubation at 18 °C for 17.5 h. Cells were harvested at 8,200 rpm for 5 min at 4 °C, resuspended in lysis buffer (20 mM Hepes, 500 mM NaCl, pH 7.4), and lysed under high pressure. Cell debris was removed by centrifugation at 8,200 rpm for 1 h at 4°C.

The supernatant was purified using a Ni-NTA column and eluted with buffer B1 (20 mM Tris, 500 mM NaCl, and 500 mM imidazole). Further purification of pE146L-ΔTM was performed by gel filtration using a HiLoad 16/60 Superdex 200 column (GE Healthcare, USA) equilibrated in buffer B2 (20 mM Hepes, 200 mM NaCl, pH 7.4). Highly purified fractions were concentrated to 6 mg/mL with 3 kDa molecular weight cutoff concentrator (Millipore) for crystallization. Proteins were flash-frozen in liquid nitrogen and stored at −80°C.

### Protein-lipid overlay assay

Commercial lipid strips (100 pmol lipids per spot) were blocked with PBST containing 3% (wt/vol) BSA for 1 h at room temperature (RT). Purified protein (6 μg/mL) was incubated with the lipid strips in PBST-BSA for 1 h, followed by three washes with PBST (5 minutes each). Anti-His mouse antibody (1:5,000; Proteintech) was applied for 1 h in PBST-BSA, washed three times with PBST, and detected using ECL Prime Western blot reagents (GE Healthcare, United Kingdom) (57).

### Crystallization and data collection

Crystallization of ASFV pE146L-ΔTM was achieved using the sitting-drop vapor diffusion method at 20°C. The best crystals were obtained by combining 1 μL protein solution with 1 μL reservoir solution (0.04 M sodium citrate dihydrate, pH 3.9, 5% wt/vol polyethylene glycol 3350; Hampton Research, USA). Crystals were cryoprotected with increasing concentrations of glycerol (5%, 15%, and 25% vol/vol), flash-frozen in liquid nitrogen, and stored.

### Structure determination

X-ray diffraction data were collected at beamline BL17U1 of the Shanghai Synchrotron Radiation Facility (SSRF) using a Pilatus 6M detector at a wavelength of 0.97918 Å. Diffraction images were integrated and processed using the HKL-2000 program. Structures were solved by molecular replacement using PHASER (58), with AlphaFold predicted models as initial templates. Model rebuilding was performed with COOT (59) and refined using Phenix. Images were generated using PyMOL.

### Liposome preparation

POPC, POPA, and cholesterol were dissolved in chloroform to a final concentration of 10 mg/mL and dried into a lipid film under nitrogen flow. Lipid films were rehydrated with buffer (200 mM NaCl, 20 mM Tris-HCl, pH 7.5) and vortexed to create a lipid suspension at a final concentration of 10 mM. Prepared liposomes were stored at 4°C for short-term use (47, 60).

### Co-sedimentation assay

Three milligrams of protein were centrifuged at 16,000 *× g* for 20 min at 4°C to remove nonspecific aggregates. Freshly prepared MLVs in different proportions were added to the supernatant and incubated at 20 °C for 30 min or at 4 °C overnight. The reactions were centrifuged at 16,000 *× g* for 20 min at 4 °C. Supernatants and pellets were separated and run on 15% SDS–PAGE. Band intensities of the pelleted protein with MLVs were quantified using ImageJ. Each experiment was performed three times, and the mean and standard errors of the measurements were reported (47, 61)

### Transfection and infection

HEK293T or WSL cells were grown to 80% confluency in a medium containing 10% FBS and transfected using JetPRIME (Polyplus) reagent according to the manufacturer’s instructions.

For viral infections, PAMs or WSL cells were infected with ASFV and incubated for 2 h at 37°C with 5% CO_2_ to allow virus attachment. The supernatants were removed and replaced with a fresh medium, followed by incubation at 37°C in 5% CO_2_ for the indicated times. To inhibit ASFV late gene expression and morphogenesis, the DNA replication inhibitor AraC (50 μg/mL, Sigma-Aldrich) was added to the media after the adsorption period (2 h) and maintained throughout the infection/transfection experiments. At the end of the transfection or infection period, the cells were fixed for immunofluorescence or EM analysis or dissociated for immunoblotting or RT-qPCR according to the experimental purpose.

### RT-qPCR

Total RNA was extracted from cells using an RNA-easy mini kit (Vazyme) according to the manufacturer’s protocols. The isolated RNA was reverse transcribed into cDNA using a PrimeScript RT Reagent Kit with gDNA Eraser (TaKaRa) in a total volume of 40 μL. Quantitative reverse transcription-PCR (qRT-PCR) was performed.

Target genes were amplified using a reaction system containing SYBR Green Mix (Bio-Rad, USA; 5 μL), primers specific for the target gene (5 μL), and double-distilled H₂O (3 μL) in a final volume of 10 μL. PCR amplification was carried out using a CFX96 Real-Time PCR Detection System (Bio-Rad, USA) with the following conditions: 15 min at 95 °C, followed by 39 cycles of 10 s at 95 °C and 30 s at 60 °C. Relative expression levels were calculated using the 2^−ΔΔCt^ method with GAPDH as the normalization control. Primer sequences are listed in Table S1.

### Immunostaining and confocal microscopy

Transfected cells were washed with PBS 24 h post-transfection, fixed with 4% paraformaldehyde at room temperature for 30 min, and permeabilized with 0.3% Triton X-100 for 10 min. After three PBS washes, cells were blocked with PBS containing 5% bovine serum albumin (BSA) for 1 h.

Cells were then incubated with primary antibodies at 37 °C, followed by Alexa-labeled secondary antibodies (Invitrogen, A-11005; 1:1,000). Nuclei were stained with DAPI (Sigma, D9542) at room temperature for 5 min. Images were captured using a confocal microscope (Zeiss LSM 800) with a 63× oil immersion objective and analyzed using ZEN Microscopy Software (Zeiss).

For infection experiments, cells were washed with PBS at the indicated times post-infection, and the same immunostaining protocol was applied. Primary antibodies included ASFV E146L (a rabbit anti-ASFV E146L protein polyclonal antibody prepared in-house), p72 (a mouse anti-ASFV p72 protein polyclonal antibody), and p30 (a mouse anti-ASFV p30 protein polyclonal antibody). Samples were imaged using a fluorescence microscope (Olympus-IX73) or a laser scanning confocal microscope (Nikon).

### Purification of ER

HEK293T cells were transfected with pCAGGS-I73R-EGFP or pCAGGS-E146L-EGFP. At 36 h post-transfection, cells were collected, and the ER fraction was isolated using an endoplasmic reticulum isolation kit (Solarbio, EX2690). The purity of the fractions was confirmed by immunoblotting for calreticulin (ER marker).

### Transmission electron microscopy

Overexpressed HEK293T cells were washed three times with precooled PBS, and 1 mL of 2.5% glutaraldehyde (Servicebio) was added to fix the cells for 30 min at room temperature. Fixed cells were transferred to 2-mL centrifuge tubes. TEM samples were performed by Servicebio.

For immuno-electron microscopy (IEM), PAMs were infected with ASFV at an MOI of 5 for 18 h. Infected cells were fixed with 2.5% glutaraldehyde (Servicebio) for 30 min at room temperature. Immunolabeling was performed using rabbit anti-ASFV E146L antibodies prepared in-house. Servicebio processed samples for imaging. Images were captured using the HZAU TEM platform (HITACHI, H-7650).

### Molecular docking

AutoDock Vina for molecular docking, which adopted a semi-flexible docking mode. To assess free binding energies, AutoDock Vina was used for the docking process. The receptors and ligands were pretreated with hydrogenation using Auto Dock Tools 1.5.7 before docking, and the Grid Box was set with the original ligand as the center. Finally, we used the Discovery Studio (2024) and PyMOL to visualize and evaluate the interactions of typical docking data. The minimum binding energy among each pair of complexes was calculated. The binding energy value is inversely proportional to the binding ability, with binding energy ≤ −5 kcal/mol signifying strong binding activity.

### Statistical analysis

Unless otherwise indicated, the data are representative of at least three independent experiments, and values are given as the mean of triplicate samples± standard deviation (SD). An unpaired two-tailed t-test was used to determine the statistical significance and performed in GraphPad Prism version 8.0.0 (GraphPad Software, San Diego, California, USA). Data are presented as the mean ± SD of three independent experiments. *P *≥ 0.05 was considered statistically non-significant (ns); *P* < 0.05 was considered statistically significant (* *P*< 0.05, ** *P*< 0.01, *** *P*< 0.001, **** *P*< 0.0001).

## Data Availability

Structural data were deposited in the RCSB Protein Data Bank (PDB) under accession code 9J46. The data that support the findings of this study are openly available in this article and are available from the corresponding author upon request.

## SUPPLEMENTAL MATERIAL

Supplemental material jvi.00834-25-s0001.docx Fig. S1 to S3; Table S1.

Movie S1 jvi.00834-25-s0002.avi ASFV pE146L is spatially co-located with the ER.

Movie S2 jvi.00834-25-s0003.avi ASFV pE146L is spatially co-located with the ER.

ASM does not own the copyrights to Supplemental Material that may be linked to, or accessed through, an article. The authors have granted ASM a non-exclusive, world-wide license to publish the Supplemental Material files. Please contact the corresponding author directly for reuse.
