# Supplementary material for: ASFV pE146L-induced ER remodeling is essential for viral replication
Source: J Virol. 2025 Aug 6;99(9):e00834-25. doi: 10.1128/jvi.00834-25 (PMC12455986; doi:10.1128/jvi.00834-25)
Supplement: Supplemental material — Fig. S1 to S3; Table S1. [file jvi.00834-25-s0001.docx]

**Supplementary Information**


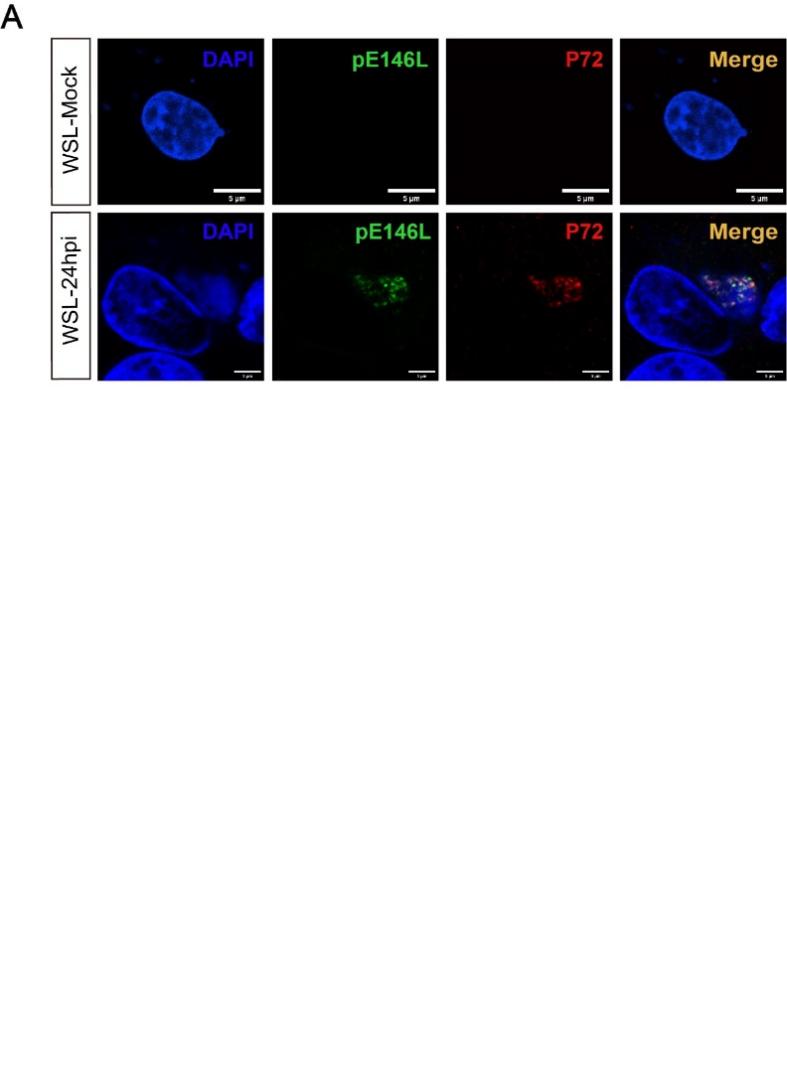


**Fig. S1** (A)Subcellular localization of pE146L in the ASFV-infected WSL cells. ASFV-infected WSL cells were fixed at 24 hpi and immunolabeled with home-made rabbit anti-pE146L (green) and mouse anti-p72 (red). Bars, 5µm.


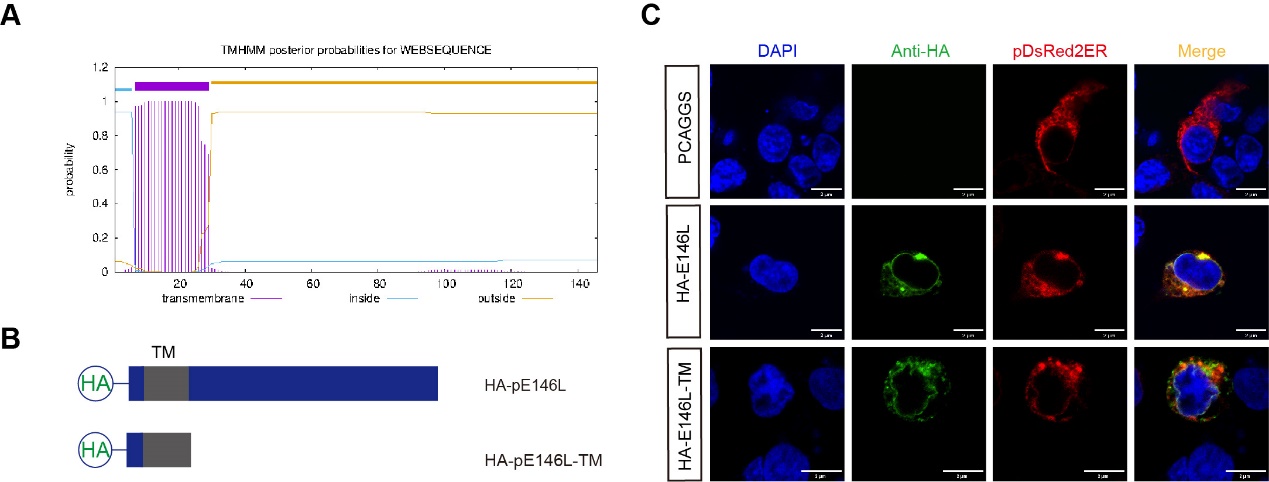


**Fig. S2** (A)The sequence of pE146L amino acids was used for predicting protein transmembrane helices by using TMHMM 2.0 website. (B) Schematic view of pE146L and constructs used here. TM, transmembrane region. (C) Immunoﬂuorescence of intracellular pE146L and pE146L-TM. 293T cells co-transfected with plasmids encoding pE146L、pE146L-TM and pDsRed2ER. Scale bars, 2 μm.


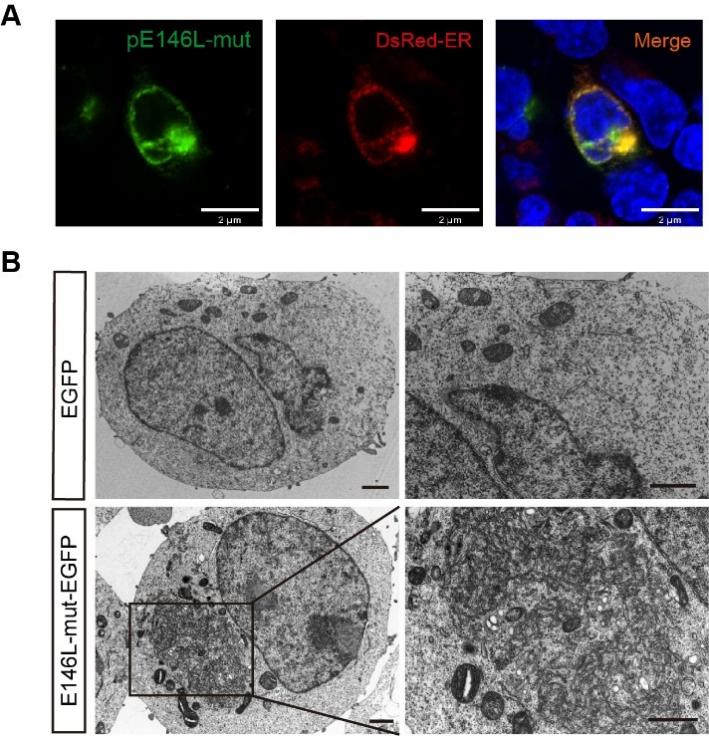


**Fig. S3** (A) Immunoﬂuorescence of intracellular pE146L-mut. 293T cells co-transfected with plasmids encoding pE146L-mut-EGFP (lipid-binding residue mutation) and pDsRed2ER. Scale bars, 2 μm. (B) Transmission electron microscopy (TEM) revealed ER morphology in pE146L-transfected 293T cells transfected indicated plasmids. Scale bars, 1 µm.

**Movie S1 and S2:**

ASFV pE146L is spatially co-located with the ER. 293T cells co-transfected with plasmids encoding pE146L-EGFP and pDsRed2ER. Three-dimensional scanning was performed using a confocal microscope.

| **Primer Name** | **Sequence** | **Description** |
| --- | --- | --- |
| PCAGGS-HA-E146L-F | TACCCATACGATGTTCCAGATTACGCTGAATTCATGGGCGGCACTACAGAC | For the gene E146L |
| PCAGGS-HA-E146L-R | AAGATCTGCTAGCTCGAGCTAAATAATACGCTGTAGTCCGGT | For the gene E146L |
| PCAGGS-HA-E146L-TM-F | TACCCATACGATGTTCCAGATTACGCTGAATTCAGGGCGGCACTACAG | For the gene E146L-TM |
| PCAGGS-HA-E146L-TM-R | TTAATTAAGATCTGCTAGCTCGAATTATACCAAATGAAAGCAATAATAATGAAGG | For the gene E146L-TM |
| PET42b-E146L-ΔTM-F | GGTGGTGGTGGTGCTCGAGATGTTTACCGGATGGTCCC | For expression of the recombinant protein |
| PET42b-E146L-ΔTM-R | GAAGGAGATATACATATGTTAATTAATACGCTGTAGTCCGGTC | For expression of the recombinant protein |
| E146L-sgRNA-F | CACCGTTGTCTATAACTATTGTGT | For construction of lentivirus sgRNA expression vector |
| E146L-sgRNA-R | AAACACACAATAGTTATAGACAAC | For construction of lentivirus sgRNA expression vector |
| PCAGGS -E146L-EGFP-F1 | GACCGGACTACAGCGTATTATTATGGTGAGCAAGGGCG | For the gene E146L-EGFP |
| PCAGGS -E146L-EGFP-R1 | CGCCCTTGCTCACCATAATAATACGCTGTAGTCCGGTC | For the gene E146L-EGFP |
| PCAGGS -E146L-EGFP-F | CATTTTGGCAAAGAATTCGCCCACCATGGGCGGCACTACAGA | For the gene E146L-EGFP |
| PCAGGS -E146L-EGFP-R | AAGATCTgCTAGCTCGAGCTACTTGTACAGCTCGTCCAT | For the gene E146L-EGFP |
| QP-GAPDH-P-F | ACATGGCCTCCAAGGAGTAAGA | RT-qPCR for pig GAPDH |
| QP-GAPDH-P-R | GATCGAGTTGGGGCTGTGACT | RT-qPCR for pig GAPDH |
| QP-CP204L-F | CGGTAGAATTGTTACGAC | RT-qPCR for CP204L |
| QP-CP204L-R | TTCTTGAGCCTGATGTTC | RT-qPCR for CP204L |
| QP-B646L-F | CCACGTAATCCGTGTCCCAA | RT-qPCR for B646L |
| QP-B646L-R | GATGATCCGGGTGCGATGAT | RT-qPCR for B646L |

**Table S1. Primers used in this study**
